# Supplementary material for: The causal relationship between the human gut microbiota and pyogenic arthritis: a Mendelian randomization study
Source: Front Cell Infect Microbiol. 2024 Nov 26;14:1452480. doi: 10.3389/fcimb.2024.1452480 (PMC11629706; doi:10.3389/fcimb.2024.1452480)
Supplement: Supplementary file 1 [file DataSheet1.zip › mendelian test/ebi-a-GCST90027568.csv_forest.pdf]

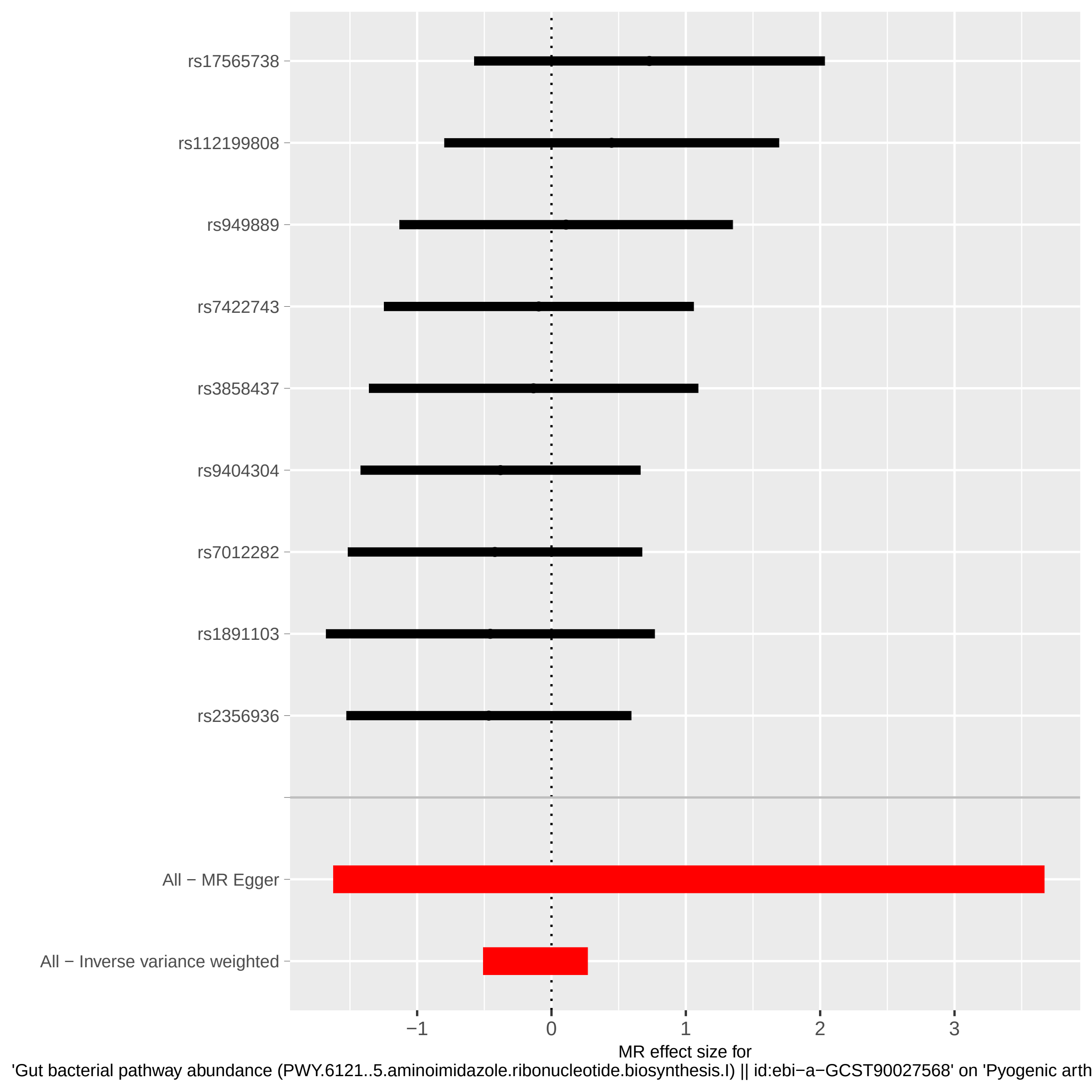
rs17565738
rs112199808
rs949889
rs7422743
rs3858437
rs9404304
rs7012282
rs1891103
rs2356936
All − MR Egger
All − Inverse variance weighted
−1
0
1
2
3
MR effect size for
'Gut bacterial pathway abundance (PWY.6121..5.aminoimidazole.ribonucleotide.biosynthesis.I) || id:ebi−a−GCST90027568' on 'Pyogenic arth
